# Dissociating Two Stages of Preparation in the Stop Signal Task Using fMRI

**DOI:** 10.1371/journal.pone.0130992

**Published:** 2015-06-25

**Authors:** Andre Chevrier, Douglas Cheyne, Simon Graham, Russell Schachar

**Affiliations:** 1 University of Toronto, Institute of Medical Science. Toronto, Ontario, Canada; 2 University of Toronto, Institute of Biomaterials and Biomedical EngineeringDepartment of Medical Imaging, Toronto, Ontario, Canada; 3 Sunnybrook Health Sciences Centre, Toronto, Ontario, Canada; 4 The Hospital for Sick Children, Psychiatry research, Toronto, Ontario Canada; Brain and Spine Institute (ICM), FRANCE

## Abstract

Often we must balance being prepared to act quickly with being prepared to suddenly stop. The stop signal task (SST) is widely used to study inhibitory control, and provides a measure of the speed of the stop process that is robust to changes in subjects’ response strategy. Previous studies have shown that preparation affects inhibition. We used fMRI to separate activity that occurs after a brief (500 ms) warning stimulus (warning-phase) from activity that occurs during responses that follow (response-phase). Both of these phases could contribute to the preparedness to stop because they both precede stop signals. Warning stimuli activated posterior networks that signal the need for top-down control, whereas response phases engaged prefrontal and subcortical networks that implement top-down control. Regression analyses revealed that both of these phases affect inhibitory control in different ways. Warning-phase activity in the cerebellum and posterior cingulate predicted stop latency and accuracy, respectively. By contrast, response-phase activity in fronto-temporal areas and left striatum predicted go speed and stop accuracy, in pre-supplementary motor area affected stop accuracy, and in right striatum predicted stop latency and accuracy. The ability to separate hidden contributions to inhibitory control during warning-phases from those during response-phases can aid in the study of models of preparation and inhibitory control, and of disorders marked by poor top-down control.

## Introduction

Preparation is important to the accurate and rapid execution of any wilful behaviour, including the ability to stop ongoing behaviour, referred to as inhibitory control. Inhibitory control involves stopping actions that are inappropriate or are no longer required, and is crucial to goal-directed behaviour in changing conditions [1]. Inhibitory control has been studied extensively using the stop signal task (SST) [2]. SST trials begin with a brief warning stimulus signalling that a trial is about to begin, followed by a choice-response stimulus. Occasionally (*e*.*g*. 33%) and unpredictably, a stop signal follows the go signal, indicating that the subject should not make that response. The latency of the stop process (stop signal reaction time (SSRT)) is estimated by subtracting the mean stop delay from the mean go reaction time. Although models of inhibitory control rely heavily on the independence of go- and stop-processes [2], it has been shown that SSRT is affected by the level of preparation [3], and by preparatory brain activities [4]. This apparent contradiction has arisen partly because the independence of going and stopping in models of inhibitory control refers only to the relative finishing times of go- and stop processes, which are modelled as being initiated by go- and stop-signals, respectively. The independence of the relative finishing times of go- and stop-processes has been interpreted to mean that these processes do not begin until after the presentation of go- and stop signals, when the race model is in fact silent about the onset of these processes. The assumption that go- and stop-processes do not begin until the presentation of go- and stop-stimuli appears to be true in the modality-specific networks that ultimately carry out or inhibit responses [5,6]. However, from the moment of the earliest warning stimuli that precede stop signals, central structures must engage and maintain top-down control over modality-specific networks until a decision is made to go or to stop [7]. Here we attempt to gain a better view of the hidden factors that affect the decision to go or stop while top-down control is being engaged, before any stop signal appears.

The warning stimuli used in the SST are brief (500 ms) compared to the temporal resolution of fMRI (e.g. 2s). As a consequence, previous studies have used indirect methods to infer preparation-related activity in stop tasks. For example, Chikazoe et al [4] included trials in which it was known that no stop signal would appear. By subtracting activity on these trials from go-trials that might include stop signals, they found activity in inhibition-related structures, suggesting that subjects were pre-activating these regions in anticipation of potential stop signals. Therefore there is strong evidence that inhibition-related activity comes into play before stop signals appear. Here we attempt to capture these preparatory activities directly and use regression analyses to assess their effects on go- and stop-processes.

Other approaches have contrasted compound trials in various ways and have inferred preparatory activities that affect inhibition, particularly in fronto-parietal networks and presupplementary motor area [4][6][7][8]. For example, some studies have separated preparation from inhibition by using long fore-period delays [9], and contrasting successful with failed stop trials across variable fore-period delays [7][8]. However, using long fore-period delays can be problematic because this kind of preparation cannot be maintained for more than a few seconds before resetting working memory [1], which would invoke preparation-phase activities that are not related to preparation. Further, contrasting failed and successful stop trials confounds differences in monitoring and feedback-reinforcement processes on these trials with differences in preparation [10]. Hu and Li [8] contrasted failed and successful stop trials using two separate deconvolution analyses; one that was time-locked to warning stimuli and one that was time locked to response stimuli. However, warning- and response phase activities are not statistically independent and so should be separated simultaneously in a single deconvolution analysis. In this study, we accomplish such a simultaneous deconvolution by capturing response-phase activities using separate regressors that were time-locked to left- and right-hand responses instead of a single regressor time-locked to the presentation of response-stimuli.

Another approach has been to present response stimuli *after* stop signals in order to isolate the circuits involved in preparing to stop a response that the subject already knows will have to be stopped [6]. However, this cannot reflect the kind of preparedness to stop that occurs before stop signals appear. We present a method to generate a whole-brain view of two phases of activity that affect our preparedness to stop when it remains uncertain whether the response on that trial will have to be stopped or not. Imaging preparation to stop in the context of uncertainty is important because we often do not have advance notice of the need to stop in real-life situations. The central decision to stop or to go is an important part of overall inhibitory control and in certain circumstances, or in certain disorders, may be more important than the modality specific skeleto-motor mechanisms that ultimately carry out or inhibit the overt response.

The value of directly separating preparation from other within-trial processes, instead of contrasting compound trials, was demonstrated in a recent study of attention deficit hyperactivity disorder (ADHD). ADHD is associated with inhibitory control deficits, and ADHD subjects have been shown to under-activate the inferior frontal gyrus (IFG) on stop trials [11][12][13]. The natural interpretation of under-activation of the IFG on stop trials has been that it reflects the under-performance of inhibitory control in ADHD, and that it manifests during the act of stopping a response. However, by separating activities before and after stop signals that had been combined in previous studies, it was demonstrated that this apparent under-activation is in fact the result of ADHD subjects deactivating the IFG before stop signals appeared, when healthy control subjects were pre-activating the IFG [14]. Contrary to previous assertions, ADHD subjects were not under-activating the IFG when stopping their responses; IFG differences in ADHD instead reflect a dysfunctional attempt to pre-activate the IFG during preparation. Therefore, rather than simply reflecting the narrative of poor inhibitory control in ADHD, the direct separation of within-trial activities suggests a dysfunctional reinforcement mechanism that tunes appropriate networks in an inappropriate way. This vital kind of distinction can contribute to the understanding and treatment of cognitive dysfunction, and could not have been found by the conventional approach of contrasting compound trials based on cognitive models of inhibitory control. Therefore, directly separating preparation from other within-trial activities can provide insights into the neural mechanisms of behavioral deficits that might be beyond the predictions of cognitive models.

Here we present an approach to separate activity that occurs after warning stimuli from activity that occurs during response phases that immediately follow. Both of these phases precede stop signals, and both of these phases should affect inhibition [3,4,6–8,15]. We used separate regressors for warning stimuli and for left- and right-hand responses, and employed a temporal jittering strategy that optimized the separability of event-types in the deconvolution analysis (as in [16,17]). Despite the brief nature of preparation in the SST, it is possible to separate rapid sequences of activity as long as they do not always co-occur, and as long as the component processes these activities reflect unfold in a known temporal order [18]. The regressors used here, reflecting warning- and response-phases in the SST, undoubtedly unfold in a known sequence in time, and do not always co-occur because one sixth of trials were successful stop trials, which contained warning stimuli but no overt response. However, although stop trials contain no response, they should contain response-phase preparatory activity even if no response regressor is used for these trials in the deconvolution analysis. To this end, we showed in a previous study that in the proposed approach, response-phase activity on successful stop trials is captured by the stop stimulus, along with activities reflecting response cancellation [17]. Further, despite the brief temporal separation between warning stimuli and responses (~ 1s), a similar approach in a previous study isolated prediction error deactivations in an ascending dopamine pathway [16] which only last 100 ms, an order of magnitude shorter than the preparatory periods being investigated here. Therefore the current approach should be capable of separating activity during warning-phases from those during response-phases that follow.

In this study, we will refer to activities related to warning stimuli as “warning-phase” activities, and activities related to responses as “response-phase” activities. Significant warning- and response-phase activities were correlated with individual differences in the speed and accuracy of going and of stopping to identify their distinct influences on inhibitory control. Despite the overall independence of the finishing times of go- and stop-processes in the SST, we expect that distinct preparatory activities during warning- and response-phases will have distinct influences on the speed and accuracy of go- and stop-processes.

Given the close temporal proximity of warning- and response-phase activity, and the fact that response-phase activities should also be present on stop trials that contain no response regressor, it is important to first determine whether the patterns of activation found here are consistent with what would be expected based on previous research using event-related potentials (ERP). Several differences between warning- and response-phases that have consistently been observed using ERP should also be present here if the current approach were successful. Firstly, warning-phases tend to activate left parietal regions, whereas response phases activate right parietal regions [19][20]. Secondly, primary motor cortex deactivates during warning phases and activates during response phases [21]. Thirdly, warning-phases primarily activate posterior regions, followed by fronto-posterior activities during response-phases [22]. Fourthly, preparation requires contributions from subcortical structures, but begins cortically [23]. Therefore, if the current approach successfully separated these phases of activity, then the results should involve: left followed by right parietal activity, deactivation followed by activation in primary motor cortex, posterior activity followed by fronto-posterior activity, and cortical activity followed by cortical-subcortical activity. After whole-brain correction, the current results contained all of these expected patterns of activity, providing a high degree of confidence that the approach can indeed separate warning- from response-phase activity. Regression analyses revealed distinct warning- and response-phase activities that had distinct influences on inhibitory control.

## Materials and Methods

### 2.1 Subjects and task design

**Fig 1:**
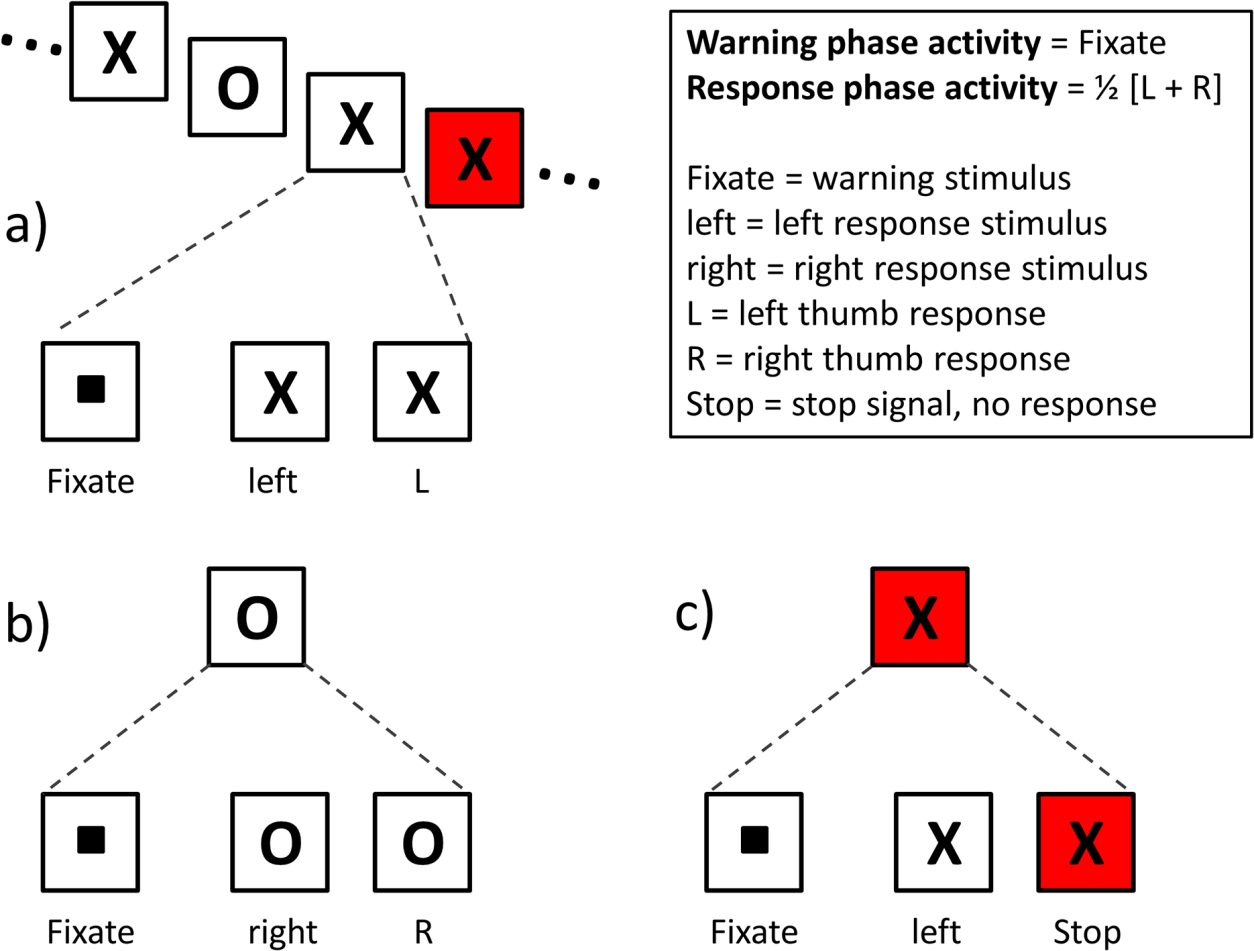
Trial types and regressors for isolating preparatory activity in the SST. **Trials began with a fixation dot (warning stimulus) in the middle of the screen for 500 ms followed by a response stimulus for 1 s.** The response stimulus was either an “X”, indicating that the subject should press a button with their left thumb (a), or an “O”, indicating that the subject should press a button with their right thumb (b). On one third of trials (c), the response stimulus was followed by a stop signal (screen colour change from black to red), indicating that the subject should not respond on that trial. The box at the top right of the figure describes the stimuli that are used to estimate warning and response phase activities.

Fourteen healthy, right-handed subjects (mean age 29.4 years) participated in the study. Subjects had normal vision and reported no medication use, medical illness or psychological problems. Subjects gave informed written consent to participate in the study, which was approved by our institutional research ethics board. Stop task trials began with a 500 ms fixate (warning) stimulus followed by a go (response) stimulus that remained on the screen for 1000 ms (Fig 1). The go (response) stimulus consisted of either the letter “X,” which indicated that the subject should make a button press with their left thumb, or the letter “O,” which indicated that the subject should make a button press with their right thumb. On one third of trials, the response-stimulus was followed by a stop signal (change in background colour from black to red), indicating that the subject should try to stop their response on that particular trial. Stop signals followed response-stimuli after an adaptive delay that ensured subjects could only successfully stop on approximately half of the stop trials presented [2]. The initial stop delay was 250 ms, and increased or decreased by 50 ms when subjects succeeded or failed to stop, respectively.

A blank screen appeared during the inter-trial interval (ITI), which was either 1 or 2 seconds, resulting in trial lengths of 2.5 or 3.5 s. The ITI’s were jittered with random combinations of spread-spectrum binary coding sequences. These binary sequences are mutually orthogonal, which maximises the diversity of temporal offsets from one trial to the next. Maximizing the diversity of temporal offsets of subsequent trials serves to maximize the number of independent equations in the deconvolution analysis, which enhances the separation of event types in the model. This timing also ensured that warning-stimuli and responses to go-stimuli only occurred in the same fMRI time-point on about half of trials. Further, although every trial contains a warning-stimulus followed by an imperative response-stimulus, not every trial contains a response (i.e. one out of six trials were successful stop trials), resulting in sufficient contrast over the course of the experiment to separate activity related to warning-stimuli from activity related to responses to go-stimuli. A 17.5 second rest followed every fourteenth trial in order to generate a reliable estimate of baseline activity. The task was performed in the MRI environment for a total of 21 minutes and 40 seconds, yielding a total of 322 trials.

### 2.2 Behavioral measures

Mean reaction times on successful go trials were calculated for all subjects, as were percent accuracy for both go trials and stop trials. The latency of the stop process is not directly observable in the SST because there are no responses on successful stop trials. However, the stop signal reaction time (SSRT) can be estimated by subtracting the mean stop delay from the mean go reaction time [2]. The SSRT is a reliable measure of inhibitory control, with shorter SSRT’s indicating faster inhibition than longer SSRT’s.

### 2.3 fMRI acquisition and subject-level analysis

Imaging was performed with a GE LX 1.5T MRI scanner (General Electric, Milwaukee, LX hardware and software platform). Anatomical data were acquired with a standard high-quality SPGR sequence (96 slices, 1.5 mm thick, FOV = 20 cm, 256x256 matrix). Functional data were collected using a spiral gradient echo pulse sequence (TE/TR/θ = 40/2000/90). Functional acquisitions were 24 slices, 6 mm thick, FOV = 20cm, reconstructed at 64x64 pixel resolution resulting in a final voxel size of 3.125 x 3.125 x 6 mm^3^. Subjects’ responses were collected using a Lumitouch fibre-optic button box (Lightwave Medical, Burnaby, British Columbia, Canada) interfaced to a laptop running the stop task paradigm at the MRI system console.

Functional data were analyzed using AFNI [24]. Images were motion corrected using a standard coregistration algorithm [25]. None of our subjects moved more than 2mm over the course of the scan, and could therefore all be included in subsequent analyses. A general linear model of stimulus vectors convolved with the hemodynamic response function (HRF) was used in the deconvolution analysis. Estimates of baseline and linear drift were generated simultaneously with 6-point HRF’s (12 second duration) for each event type outlined below (HRF delay = 2TR = 4 seconds).

The following event types were included in the deconvolution analysis: Fixate = warning phase; X = left hand response; O = right hand response; Stop = successful inhibition; Fail = error processing on failed inhibition trials. Go-trials were modelled using Fixate and response (X or O) events. Stop trials were modelled using Fixate and Stop events. Failed stop trials were modelled using warning (Fixate), response (X or O) and error processing (Fail) events. Fig 1 portrays the relevant trial types and regressors used to separate warning from response phase activity. Warning-phase activity was isolated with the Fixate map. Response-phase activity was isolated using the contrast ½(X+O), which enhanced activities that would be common to both responses, while suppressing lateralized activity related to the handedness of the response (as in [17]).

Multicollinearity of a deconvolution analysis can be optimized by minimizing the inverse average error of the model matrix, which in the current experimental design was below 10^−15^. Further, the inherent correlation between warning- and response-phase regressors can be computed prior to data collection by determining the angle between them using the formula for the dot product (i.e. θ = cos^-1^ [(Σ_i_ warning_i_ * response_i_) / |warning| * |response|], where i ranges over all time fMRI time points, warning_i_ and response_i_ equal 1 when the event occurs during time point i and 0 when it does not occur, and |x| denotes the magnitude of vector x). A grazing angle (near 0) signifies high collinearity whereas angles approaching orthogonality (i.e. 90°) signify low collinearity and a basis for meaningful contrast. Using this method we found that the angle between warning- and response-phase regressors was 62.8°, corresponding to a correlation of around 0.5. Using this value, we were able to quantifiably estimate whether warning- and response-phase activities can be separated. We found the detection-tolerance (calculated as 1-r^2^) to be 0.75. Tolerance values less than 0.1 or 0.2 would indicate that multicollinearity is a problem. It appears the current approach should be capable of separating warning- from response-phase activity as the tolerance is nearly a factor of 4–8 times better than required. From a qualitative perspective, when regressors are multicollinear, the respective maps generally show activation and deactivation in the same anatomical locations. If the approach was successful, then regions that are known to activate during both of these phases (e.g. primary visual areas) should exhibit positive activity in both of these maps, whereas regions that are known to deactivate on the presentation of warning-stimuli and then activate on the presentation of response-stimuli (e.g. primary motor cortex) should show deactivation followed by activation. Therefore as a final test of the multicollinearity between warning- and response-regressors, their unthresholded group activation maps were visually inspected to determine if this was the case (S1 Fig Warning-phase and S2 Fig Response-phase). The low multicollinearity between regressors despite their close temporal proximity was due to the experimental design: firstly, temporal jittering using spread spectrum binary coding sequences maximized the number of independent equations in the deconvolution; secondly, one out of six trials (successful stops) contained fixate cues, but no overt response (X or O); thirdly, the interplay of 2.5 and 3.5 s trial lengths with a TR of 2 s ensured that warning- and response-regressors did not always occur in the same fMRI time-point even if they both occurred in the same trial.

Intensity maps for all event types, and the ½(X+O) contrast were generated for each subject by taking the area under the HRF, estimated by the sum of HRF coefficients generated by AFNI’s 3dDeconvolve program. These maps were then warped into Talairach space, and blurred (6mm FWHM).

### 2.4 Group-level analysis

Activity maps reflecting warning- and response-phases (Fixate and ½(X+O) maps) for all subjects were entered into a group ANOVA to determine activities that were common across the group. This ANOVA output (distributed as a t* statistic with 65 degrees of freedom due to the number of subjects in the study, and the number of event-types in the model) was then cluster/thresholded according to Gaussian field theory using AFNI’s AlphaSim program in order to correct for multiple statistical comparisons as in [17]. This analysis required that significant voxels be part of a larger cluster of at least four original voxels (234.4 mm^3^) with a minimum z-score of 3.3 for an overall α < 0.05. Therefore every region that significantly activated has an effect size greater than 3.3. These regions represent statistically significant (whole-brain corrected) activations for the group. In order to test whether activities in these regions might affect the balance between going and stopping, we sorted the group of fourteen subjects according to their go reaction time, go accuracy, stop signal reaction time, and percent stop accuracy. We then performed regression analyses to identify warning- and response-phase activities that predicted individual differences in the speed or accuracy of going or of stopping.

## Results

### 3.1 Subject performance

Behavioral data were consistent with normal adults in non-MRI environments. Go responses were fast (597.7 ± 53.7 ms) and accurate (99.6 ± 0.98%), and the mean stop signal reaction time (SSRT) was within normal range (210.3 ± 48.0 ms) for adults. Approximately half of the stop trials presented contained erroneous responses (48.8 ± 2.4%), indicating that the tracking algorithm was functioning as expected. The speed of the go-process, measured by mean go reaction time, was not significantly correlated with the speed of the stop-process, as measured by the SSRT (r^2^ = 0.053; p = 0.57) in our subjects. Faster inhibitory control was therefore not simply a result of slower responses, which is consistent with the well-established independence of the relative finishing times of go- and stop-processes in the SST [2].

### 3.2 Warning- and response-phase activities

**Fig 2:**
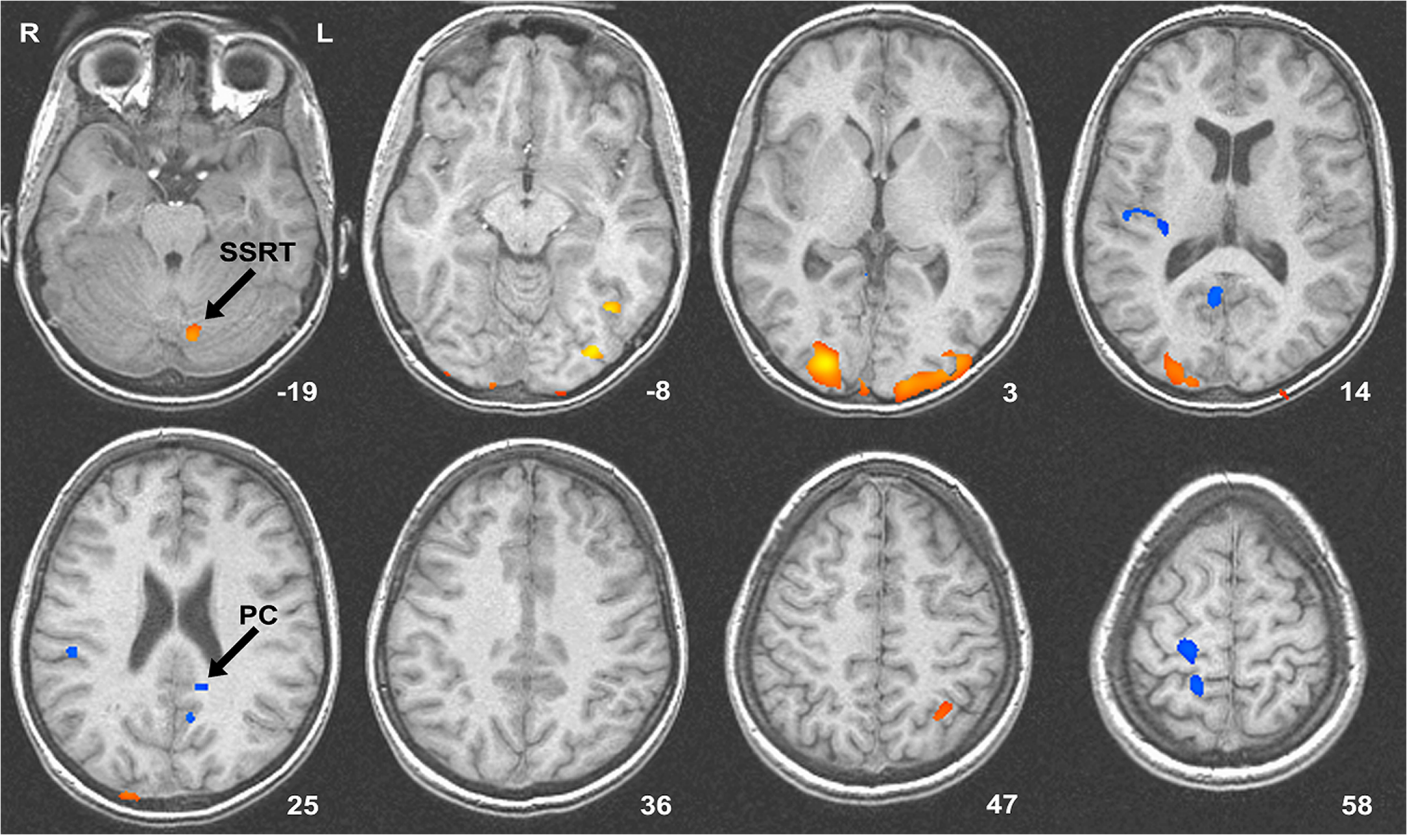
Whole-brain corrected activities during warning-phases. Arrows indicate regions where the level of warning-phase activity significantly predicted individual differences in SSRT (cerebellum), and PC (posterior cingulate BA 23/31). Abbreviations: SSRT–stop signal reaction time; PC–percent correct inhibition. Numbers in bottom right corners refer to the z coordinate in Talairach space. Increased activity is coloured red-yellow and decreased activity blue. Slices are portrayed in radiological space (right = left).

Single subject deconvolution models had a low degree of multicollinearity, as indicated by inverse average errors on the order of 10^−15^. fMRI results correspondingly showed a clear differentiation between activities during warning- compared to response-phases of the task. Visual inspection of warning- and response-phase maps revealed that regions that are known to activate during both of these phases (e.g. primary visual areas) showed positive activity in both of these maps, whereas regions that are known to deactivate and then activate (e.g. primary motor) showed negative activity in the warning-phase and increased activity during the response-phase (S1 Fig Warning-phase and S2 Fig Response-phase). Whole brain correction revealed warning-related activity changes in posterior networks that have previously been implicated in motor preparation (Table 1). Decreases in BOLD activity were seen in right primary sensory and motor regions (Fig 2), posterior cingulate and lingual gyri. Increases in BOLD activity were seen in right superior parietal cortex and cerebellum. No significant activity changes were present in prefrontal cortex, thalamus or basal ganglia. By contrast, response-phases contained significant BOLD activities in prefrontal cortex, thalamus and basal ganglia as well as in posterior cortical networks and lateral cerebellum (Table 2).

**Table 1:** List of whole-brain corrected BOLD activities during warning-phases.

Structure	BA	Location	Estimate	Correlations
L parietal	7	-29, -55, 49	3.1	-
R occipital	18	26, -86, 1	9.0	-
L fusiform	37	-37, -61, -10	9.6	-
L cerebellum (vermis)	-	-7, -73, 18	5.3	SSRT
R primary motor	4	19, -26, 58	-3.0	-
R primary sensory	3	16, -42, 63	-3.4	-
R inferior parietal	40	47, -27, 22	-3.7	-
R insula	13	49, -18, 15	-2.8	-
L posterior cingulate	31	-6, -57, 21	-4.5	-
L posterior cingulate	31	-7, -53, 31	-3.7	-
L posterior cingulate	23/31	-10, -45, 24	-2.8	PC
R posterior cingulate	23	9, -57, 15	-3.0	-

BA–Brodmann area; SSRT–regions that significantly correlated with stop signal reaction time; PC–regions that significantly correlated with stop accuracy (i.e. percent correct stop trials). Locations indicate maximum activity cooridnates for all significant clusters, and are given in Talairach coordinates. Activity estimates in arbitrary units.

**Table 2:** List of whole-brain corrected BOLD activities during response-phases.

Structure	BA	Location	Estimate	Correlations
R inferior parietal	40	42, -45, 50	6.3	-
L SMA	6	-7, 8, 60	7.1	-
L pre-SMA	6/24	-3, 1, 47	5.2	PC
L ACC	24	-10, -15, 34	4.7	-
R ACC	32	7, 32, 24	5.5	-
R superior frontal	10	20, 50, 24	6.9	PC
R superior frontal	10	30, 47, 0	13.3	RT, PC
L occipital	18	-25, -86, 3	9.4	-
R inferior temporal	20	43, -13, -28	5.6	-
R lingual	17	15, -89, -3	11.2	-
L putamen	-	-22, 10, 11	5.6	RT, PC
L globus pallidus (internal)	-	-15, -8, -4	9.1	RT, PC
R putamen	-	20, 5, 0	9.1	SSRT, PC
R thalamus (ventral lateral)	-	15, -10, 16	5.6	SSRT
L cerebellum	-	-30, -67, -28	7.5	-
R precuneus	7	-7, -65, 35	-8.2	-
L cuneus	18	0, -78, 25	-7.0	-
L superior temporal	22	-46, -21, -8	-6.0	RT, PC
R insula	21	44, -9, -7	-9.4	-
R orbitofrontal	11	2-, 12, -11	-9.1	-
L orbitofrontal	11	-25, 21, -12	-12.2	-
L medial frontal	10	-8, 59, 2	-9.8	-
R ACC	25	-1, 4, -5	-10.5	-

BA–Brodmann area; SMA–supplementary motor area; ACC–anterior cingulate cortex; RT–regions that significantly correlated with go reaction time; SSRT–regions that significantly correlated with stop signal reaction time; PC–regions that significantly correlated with stop accuracy (i.e. percent correct stop trials). Locations indicate maximum activity cooridnates for all significant clusters, and are given in Talairach coordinates. Activity estimates in arbitrary units.

### 3.3 Regression analyses

Whole-brain corrected warning-phase activities were regressed with subjects’ mean go reaction time, go task accuracy, stop accuracy and SSRT in order to determine whether activity in these regions predicted individual differences in behavior. No warning-phase activations showed a significant correlation with mean go reaction time or go task accuracy. By contrast, warning-related activity in the vermis of the cerebellum was significantly correlated with SSRT (r^2^ = 0.38; p = 0.018), indicating that greater activity in this part of the cerebellum during warning-phases predicted a longer latency of inhibition, but this region was not significantly correlated with stop accuracy. However, warning-phase activity in one part of the posterior cingulate cortex (PCC) (BA 31) was significantly correlated with stop accuracy (r^2^ = 0.27; p = 0.031), indicating that greater activation, or, more properly, *less de*activation, predicted a higher likelihood of successfully stopping on stop trials. Warning-phase activities that predicted individual differences in behavior are portrayed in Fig 2, and indicated in Table 1).

**Fig 3:**
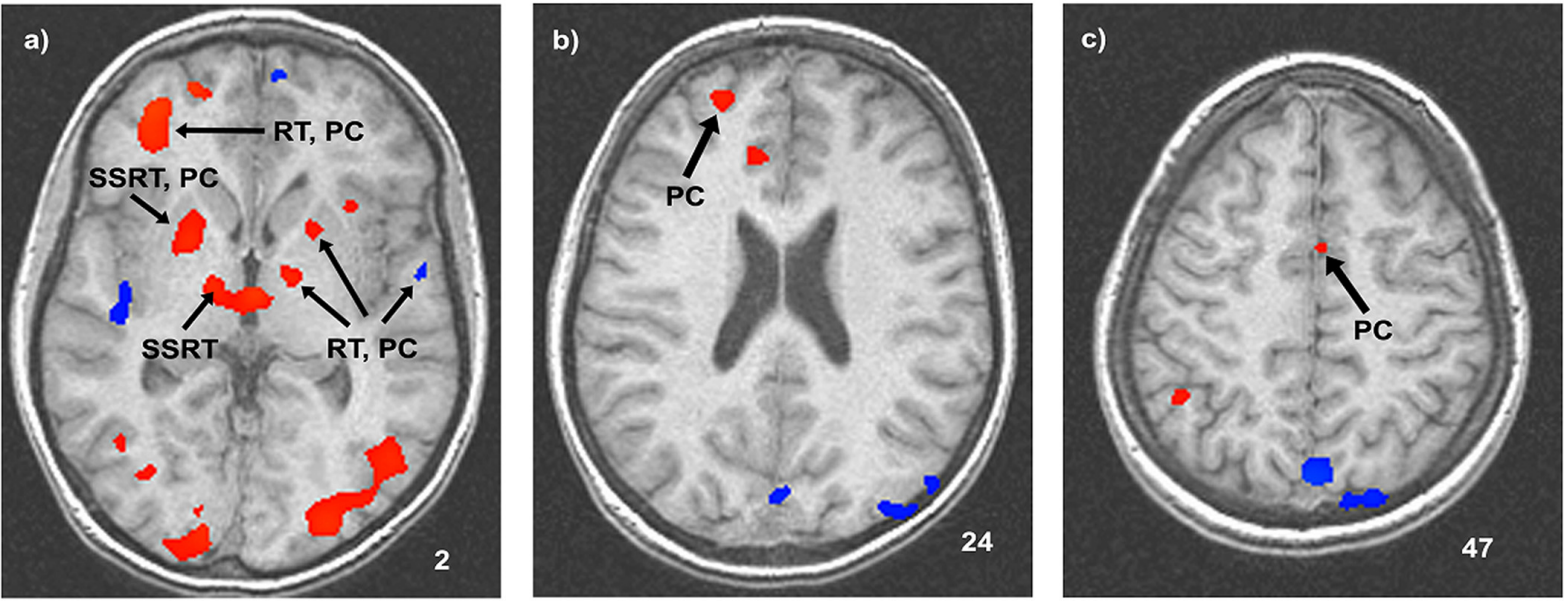
Whole-brain corrected activities during response-phases. Arrows indicate regions that significantly predicted individual differences in behaviour. a) Right superior frontal BA 10 predicted RT and PC; right putamen predicted SSRT and PC; right ventral lateral thalamus predicted SSRT; left putamen, internal globus pallidus and left superior temporal BA 22 predicted RT and PC. b) Left superior frontal BA 10 predicted PC. c) Left pre-SMA predicted PC. Abbreviations: RT–go reaction time; PC–percent correct inhibition; SSRT–stop signal reaction time.

Whole-brain corrected response-phase activities were regressed with mean go reaction time, go task accuracy, SSRT and stop accuracy to determine whether the speed or accuracy of behaviour could be predicted by individual differences in response-phase activity. Response-phase activities that predicted individual differences in behaviour are portrayed in Fig 3 and indicated in Table 2). No response-phase activities were significantly correlated with individual differences in go task accuracy. However, left putamen (r^2^ = 0.36; p = 0.013), left globus pallidus (r^2^ = 0.40; p = 0.009), left superior temporal gyrus BA 22 (r^2^ = 0.35, p = 0.015) and right superior frontal gyrus BA 10 (r^2^ = 0.24; p = 0.043) activities were significantly correlated with mean go reaction time, indicating that greater response-phase activity in these regions predicted longer response times. In addition, every region that significantly predicted go reaction time, also predicted stop accuracy (left putamen: r^2^ = 0.41, p = 0.0085, left globus pallidus: r^2^ = 0.65; p = 0.00032, left superior temporal gyrus BA 22: r^2^ = 0.61, p = 0.00059, and right superior frontal gyrus BA 10: r^2^ = 0.29, p = 0.026). Although go reaction time was strongly correlated with stop accuracy (r^2^ = 0.66, p = 0.00043), there were two regions of response-phase activity that predicted greater stop accuracy but not longer reaction times: left pre-supplementary motor area (pre-SMA) BA 6/24 (r^2^ = 0.23, p = 0.049) and right superior frontal gyrus BA 10 (r^2^ = 0.3, p = 0.026), which means that more response-phase activity in these areas predicted a higher probability of stopping on stop trials without affecting the speed of the go-process. The only response-phase activities that predicted individual differences in stop signal reaction time were in right putamen (r^2^ = 0.33; p = 0.018) and ventral lateral thalamus (r^2^ = 0.16; p = 0.019), indicating that subjects who exhibited greater response-phase activity in these areas had a longer latency of inhibition. Finally, response-phase activity in right putamen, which predicted longer stop signal reaction time, also predicted higher stop accuracy (r^2^ = 0.27, p = 0.024), whereas activity in ventral lateral thalamus did not (r^2^ = 0.2, p = 0.062).

## Discussion

The current approach revealed warning- and response-phase activities in central structures that are involved in preparing to decide whether to stop or to go, which can bring relevant modality-specific inhibition networks into play. We used a stop task that involved the presentation of warning stimuli before the presentation of imperative response stimuli, which, in turn, were occasionally followed by a stop signal. We developed an imaging approach capable of separating this sequence of activities, and applied regression analyses to identify preparatory activities that influenced go- and stop-processes before stop signals appeared. The results add detail to previous studies that have demonstrated the influences of preparation on inhibitory control in health and disease [4][6][7][26][27].

Separating these phases of activity appears to have distinguished two important stages of engaging top-down control: first signalling the need for top-down control during warning-phases, followed by the implementation of that top-down control during response-phases. The presence of activity in posterior cortical regions and cerebellum during warning-phases reflects the bottom-up call for the upcoming need to exert top-down control at the beginning of a trial. Activities in fronto-posterior and cortical-subcortical networks during response-phases that follow reflect the implementation of that top-down control, which must balance the need to respond quickly with the occasional need to stop that speeded response. The current approach identified preparatory activities that influenced inhibitory control in the same regions that have been found in previous approaches and in several regions not found in previous approaches that have not separated warning- and response-phase activities.

### 4.1 Warning- and response-phases can be separated with fMRI:

One indication of the success of the current approach is that warning- and response-phase maps contained entirely unique distributions of activity. Previous approaches that have employed separate deconvolution analyses have generated warning- and response-phase maps with greatly overlapping distributions of activity. A further indication of the success of the current approach is that the patterns of warning- and response-phase activities we found are highly consistent with observations from previous ERP studies that have not previously been distinguished using fMRI.

Several patterns of activity identified here are highly consistent with previous observations from cued-response tasks using ERP, providing a high degree of confidence that the method succeeded in separating warning- from response-phase activities. Firstly, the observation of left followed by right parietal activity has been demonstrated directly with ERP [19][20] but only indirectly with fMRI [28]. Secondly, the current finding of deactivation in primary motor cortex after warning stimuli followed by positive activity after imperative stimuli has previously been observed using ERP in cued-response tasks [21][23] but has not been distinguished with a whole-brain fMRI design. Deactivation of primary motor cortex during warning-phases that precede response-stimuli has been interpreted as inhibition of the afferent aspect of reflexive behaviour in order to ensure a more central regulation of motor output [29]. Thirdly, warning stimuli activated only neocortical regions (and cerebellum), which is consistent with previous assertions from scalp recordings in monkeys that preparation requires contributions from subcortical structures, but begins cortically [23]. Fourthly, the finding of activity in exclusively posterior networks after warning stimuli followed by fronto-posterior activities after imperative stimuli has been suggested [22] but has not previously been shown in a whole-brain context.

### 4.2 Regions that affect the balance between going and stopping—Regression analyses

Whole-brain corrected preparatory activities were correlated with individual differences in the speed and accuracy of the go- and stop-processes. Despite the independence of the finishing times of go- and stop-processes, regression analyses identified distinct warning- and response-phase activities that covertly affect distinct components of inhibitory control before stop signals appear.

A previous study by Chikazoe et al found that preparatory activities in frontoparietal regions was proportional to the preparation cost associated with longer warning-phases [4]. However, their approach combined frontal, parietal and other areas as a single region of interest, so it is difficult to determine which brain regions drove this correlation. The only other previous study that has found preparatory activities that were correlated with behavioural measures of inhibitory control stated that subjects with shorter SSRT, as determined by a median split, exhibited greater activity in orbitofrontal cortex[8]. However, this result was obtained by contrasting the difference between failed and successful stop trials from two separate deconvolution analyses; one time-locked to the warning stimulus and one time-locked to the go-signal. This result was likely driven by the fact that this part of orbitofrontal cortex strongly deactivates on failed stop trials [16], which would lead to apparently strong activation using the contrast (successful stop)-(failed stop). Although these authors did attempt to rule out error-related processing by excluding from analysis any areas that exhibited greater activity during failed than successful stop trials, this would not be the case for the orbitofrontal cortex. We show that warning- and response-phases are better separated using a single deconvolution analysis because these phases of activity are not statistically independent and should therefore be solved for simultaneously.

We found that initial warning stimuli deactivated the posterior cingulate cortex, with less deactivation predicting a less accurate stop process (i.e. lower percent successful inhibition). A previous study by Jaffard et al [7] that investigated activity associated with warning-phases also found deactivation in this part of the posterior cingulate cortex, and ascribed this activity to general alerting processes. Regardless of the general role of this structure, we show that activity here affects the accuracy of inhibitory control. This area of the posterior cingulate is part of the ‘task-negative network’. This network is known to be less anti-correlated with task-specific activity as working memory load increases [30]. Therefore subjects who deactivated this area to a lesser extent during warning-phases may have used a strategy with greater working memory load, causing a slower stop process. Warning stimuli also invoked activity in vermis of the cerebellum, where greater activity predicted a slower stop process. Given that this part of the cerebellum is involved in response readiness [31], it appears that subjects who were more oriented towards the go-component of the task required more notice in order to successfully stop. This is consistent with previous findings that varying fore-period (i.e. warning) delays affects inhibitory control by affecting response readiness [3,7].

Response-phase activity in left superior temporal gyrus BA 22 and right superior frontal gyrus BA 10 predicted longer go reaction time and higher stop-accuracy. Superior frontal gyrus serves as an interface between high level representations of externally and internally generated states, and is crucial to the ongoing evaluation of the consequences of decisions [32]. Left superior temporal gyrus projects to superior frontal BA 10 [33], and is involved in the understanding and generalizations of words, and therefore likely served to invoke a representation of the stop-rule in working memory before stop signals appeared. By contrast, pre-SMA BA 6/24, which has been associated with preparatory inhibition activity in previous studies [4][6], predicted stop accuracy, but not go-reaction time. Pre-SMA is involved in procedural learning and the development of skills requiring elaboration of motor behaviour [34]. Therefore, neocortical response-phase activities that predicted variability in stop task performance are consistent with a network that generates internal representations of task goals (superior temporal cortex), which engage top-down control (superior frontal cortex) over pre-SMA and motor circuits that in turn project to subcortical structures, where control of primary motor output can be achieved [7].

Subcortical response-phase activities in the left putamen and the internal segment of globus pallidus predicted longer go reaction time and higher stop accuracy. Activity in left putamen is associated with the retention and recall of visuomotor response mappings [35][36]. The significant correlation of activity in left putamen with go reaction time is consistent with previous observations that activity here is related to the degree of response preparation [37]. Response-phase activity in the internal segment of the left globus pallidus also predicted longer go reaction time and greater stop accuracy. The initiation of skeletomotor movements is under the inhibitory influence of the internal segment of globus pallidus [38], which might be the skeletomotor correlate of fixation neurons that have been indentified in oculomotor versions of the SST [5]. Taken together, these correlations indicate that preparatory activity in left putamen and globus pallidus serve to regulate the speed of the go-process by both preparing the go-response (left putamen) and delaying the onset of that response (globus pallidus). These results indicate that subjects who activated left striatum more strongly after imperative response-stimuli used a strategy that involved longer delays before the initiation of a response, which explains the correlation with longer response times in these subjects.

In contrast with the left striatum, in which response-phase activity predicted longer go reaction times, response-phase activities in similar structures in the opposite hemisphere (i.e. right putamen and ventral lateral thalamus) predicted longer SSRT. These areas are known to be more strongly activated under conditions that emphasize speed over accuracy [27]. Greater preparatory activity in right putamen and ventral lateral thalamus appears to reflect a strategy that emphasizes going over stopping. Therefore, despite the independence of the relative finishing times of go- and stop-processes, there are hidden preparatory processes that are involved in establishing speed-accuracy trade-off.

### 4.3 Potential applications to the study of pathological states

Recent studies of pathological populations that have investigated inhibition-related preparatory activities have found that preparatory activity is more important to the success or failure on a given stop trial than actual stop-phase activity [14][26][39]. The additional detail afforded by the current approach could therefore add insight into the nature of inhibitory control deficits in a wide array of disorders.

Several neurological and psychiatric disorders are marked by apparently similar inhibitory control deficits. The addition of neuroimaging data can help to discriminate subtle differences between these kinds of populations that are not observable from behavioural data alone, or imaging approaches that contrast compound trials based on predictions of cognitive models. This study shows two distinct phases of activity that precede the appearance of stop signals, both of which contain regions whose activity regulates distinct components of the go- and stop-processes. The current approach could therefore be used to help identify where in the brain, and at which point in a trial, activity underlying deficient inhibitory control becomes atypical, and how one disorder might differ from another despite apparently similar behavioural deficits. For example, disorders associated with poor top-down control such as ADHD and traumatic brain injury might reflect a failure to recruit top-down control, rather than a failure to implement top-down control [40]. The ability of the current approach to separate activities that recruit top-down control during initial warning-phases from those that actually implement top-down control during response-phases can be exploited to address these kinds of questions.

Regression analyses suggest candidate regions that might be differentially involved in various disorders marked by apparently similar inhibitory control deficits. For example, in disorders associated with working memory problems such as schizophrenia [41], the current approach could differentiate atypical activity associated with abnormal disengagement of the task-negative network during initial warning phases (posterior cingulate), from failure to invoke a representation of the stop rule in working memory (superior temporal) or of working memory itself (superior frontal) during response phases. Such a hypothesis could be tested by applying the current approach to a cued-response task that manipulates working memory load, such as n-back or selective stopping tasks. This kind of information is potentially useful for understanding the unique neurophysiology of various disorders, detecting individuals at risk, tracking progress of disorders, monitoring interventions, and discovering genetic and metabolic correlates of abnormal brain states.

## Conclusions

This is the first fMRI study to use a design that permits direct estimation of neural responses during brief warning- and response-phases in the SST. Our results indicate that the time course of preparing to stop begins at the onset of the initial warning stimulus, and continues up to the point of response execution or response interruption. Warning-phase activities appear to reflect signalling the need for top-down control, and response-phase activities reflect the implementation of that control. Both of these phases contained activities that affect going and stopping. The dependence of inhibitory control on distinct warning- and response-phase activities demonstrated here points to the importance of separating these activities from activities after stop signals appear when imaging inhibitory control. The ability to separate these processes with fMRI can aid in the study of top-down control deficits, especially those in which failure to signal top-down control could mimic symptoms resulting from disruptions in actually implementing top-down control, such as ADHD and traumatic brain injury.

## Supporting Information

S1 Fig Warning-phase montage. (TIF)

S2 Fig Response-phase montage. (TIF)
