# Supplementary figures and images for: Dissociating Two Stages of Preparation in the Stop Signal Task Using fMRI
Source: PLoS One. 2015 Jun 25;10(6):e0130992. doi: 10.1371/journal.pone.0130992 (PMC4481508; doi:10.1371/journal.pone.0130992)

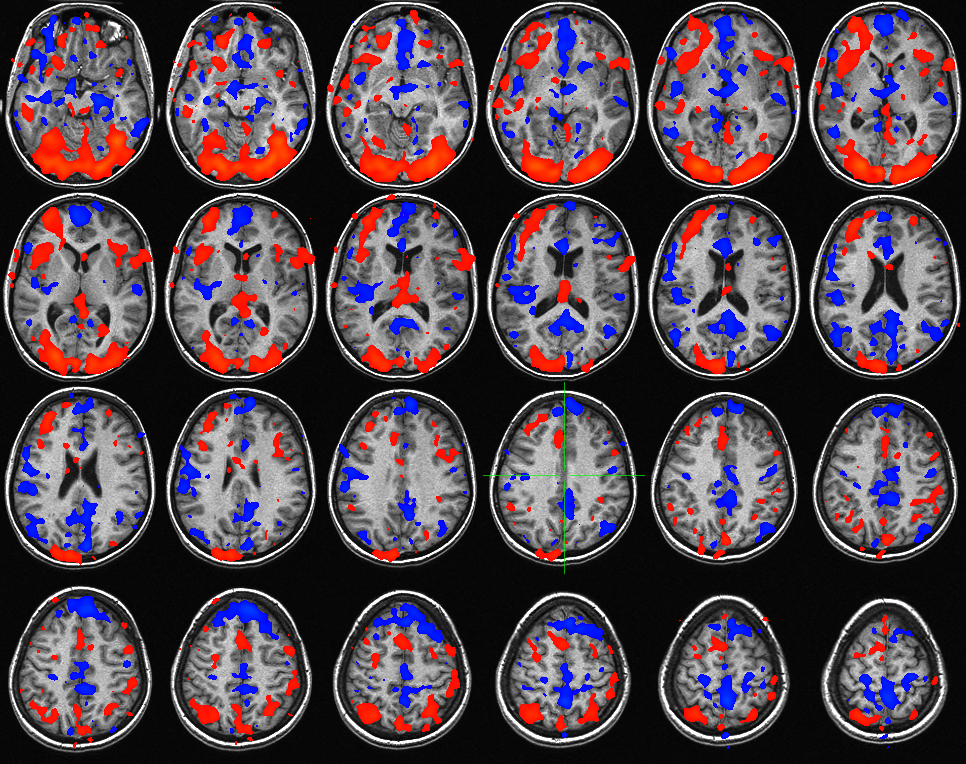

Supplement: S1 Fig — (TIF) [file pone.0130992.s001.tif]

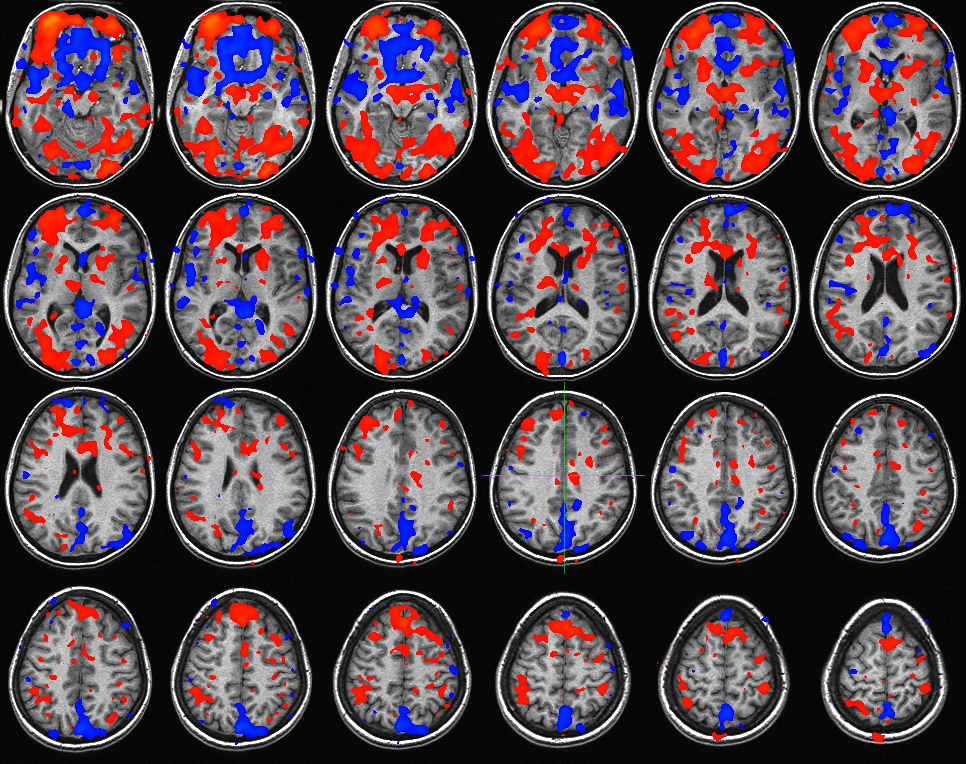

Supplement: S2 Fig — (TIF) [file pone.0130992.s002.tif]
